# Prometheus® liver therapy in children with acute liver failure

**DOI:** 10.1186/cc14461

**Published:** 2015-03-16

**Authors:** J Vande Walle, S Claus, E Snauwaert, J De Rudder, A Raes, M Dick, A Prytula, W Van Biesen, S Eloot

**Affiliations:** 1 Ghent University Hospital, Gent, Belgium

## Introduction

The Fractionated Plasma Separation and Adsorption System Prometheus® (Fresenius Medical Care, Germany) aims at being a supportive therapy as a bridge to transplantation or recovery in adults with liver failure. The system offers specific challenges when applied in children due to the large extracorporeal volume (700 to 750 ml). We therefore developed an adapted protocol for the application in children.

## Methods

Priming of the blood circuit is performed using 2 l isotonic saline, whereas the plasma circuit, containing both adsorption devices, is filled with 2 U fresh frozen plasma or 400 ml stabilized solution of human plasma proteins. Next, for children with body weight (BW) <25 kg, a solution of 60 to 65% packed cells (PC) is infused in the inlet blood line at 40 ml/minute. The volume of PC needed is calculated based on the circuit priming volume and the maximum allowed extracorporeal blood volume of the child (= 8 ml/kg × BW). After the priming phase, blood and plasma flow are increased to at least 100 ml/ minute and 200 ml/minute, respectively, and dialysate flow is set at 300 ml/minute. Regional citrate anticoagulation is done with a calcium-free dialysate, while, eventually, heparin is added to the priming solution. Post treatment, the circuit volume is either not reinfused (BW <25 kg) or reinfused using isotonic saline (BW >25 kg), with a volume depending on the hydration status and the originally infused volume of PC. Reduction ratios (RRs, %) of urea, creatinine (Crea), bilirubin (bili), and ammonia (NH_3_) were calculated from pretreatment and posttreatment serum concentrations. Primary and secondary patient outcome was evaluated.

## Results

Eight children (five male/three female), 8.6 ± 5.9 years old (range 2 to 15.6 years), BW 32 ± 21 kg, GFR 71 ± 20 ml/minute/1.73 m^2^, with an uncuffed double lumen dialysis catheter (8 to 14 Fr Femoralis (*n *= 6) and 9 Fr Jugularis (*n *= 2)) were treated according to this protocol. In total, 19 sessions were executed using FX40 (*n *= 13), FX50 (*n *= 3), and FX60 (*n *= 3) dialysers during 6.5 ± 0.9 hours. Blood flow was 149 ± 45 ml/minute, albumin flow 226 ± 49 ml/minute, and ultrafiltration flow 432 ± 517 ml. RRs were 70 ± 15% (urea), 34 ± 14% (Crea), 44 ± 16% (bili), and 36 ± 10% (NH_3_). Primary survival rate was 100%. Four patients were transplanted (bridge to transplant) of which, however, one died within 30 days after discharge from the ICU. The fifth patient died due to primary disease 9 months after treatment, and the remaining three patients fully recovered (bridge to recovery).

## Conclusion

In conclusion, this adapted Prometheus® protocol is promising for the treatment of children with liver failure.

